# Supplementary material for: Collective Variables for Conformational Polymorphism in Molecular Crystals
Source: J Phys Chem Lett. 2023 Jan 23;14(4):971–6. doi: 10.1021/acs.jpclett.2c03491 (PMC9900638; doi:10.1021/acs.jpclett.2c03491)
Supplement: Supplementary file 1 — jz2c03491_si_001.pdf [file jz2c03491_si_001.pdf]

Supporting Information: Collective Variables for Conformational Polymorphism in Molecular Crystals

Oren Elishav,[†,‖] Roy Podgaetsky,[†,‖] Olga Meikler,[‡] and Barak Hirshberg[*,†,¶,§]

†*School of Chemistry, Tel Aviv University, Tel Aviv 6997801, Israel*

‡*Rafael Ltd., P.O. Box 2250, Haifa 3102102, Israel*

¶*The Center for Computational Molecular and Materials Science, Tel Aviv University, Tel Aviv 6997801, Israel.*

§*The Ratner Center for Single Molecule Science, Tel Aviv University, Tel Aviv 6997801, Israel.*

‖*O.E. and R.P. contributed equally to this work*

E-mail: hirshb@tauex.tau.ac.il

MD simulations were carried out using LAMMPS and PLUMED2. We used a time step of $1\,fs$, which showed energy conversion within 0.1% in an NVE simulation. In simulations of isolated molecules, the cell consisted of a single molecule of 36 atoms with the initial configuration of the CIF file after minimization (conjugate gradient). Then, we collected data on the fluctuations of the improper angles at a temperature of 50K and a pressure of $1\,atm$. We used a Nose'-Hoover chains thermostat and barostat with Parrinello-Rahman full-cell fluctuations.[S1–S4] The temperature and pressure were maintained with damping parameters of $10\,fs$ and $100\,fs$, respectively. Simulation of the solid consisted of four molecules (144 atoms) with the initial geometries as in the CIF file after minimization and thermalization

of 1.1 ns. Since LAMMPS flips the simulation box, if any side length increases to 1.6 times its initial value, the cell angles can jump to complementary angles. We reversed this process when flipping occurred in all Figures for clarity. In WTMetaD simulations, upper and lower harmonic walls with a spring constant of 35 $kcal\, mol^{-1}$ were employed to improper angles 2, 3, and 5. The upper and lower values used are the minimum and maximum values observed from unbiased simulations of the isolated molecules.

Table S1: Lattice parameters of CL-20 polymorphs modeled with the SB-CL20-CCNN force field, at 300 K and 1 atm

CL-20		a(Å)	b(Å)	c(Å)	$\alpha(°)$	$\beta(°)$	$\gamma(°)$	density$(g \cdot cm^{-3})$
ϵ	Exp^a	8.852	12.556	13.386	90	106.82	90	2.044
	CL-20-CCNN FF	9.023	12.365	13.86	88.59	107.26	90.98	1.971
	dev %	1.93	1.52	3.6	1.6	0.4	1.1	3.5
β	Exp^a	9.676	13.006	11.649	90	90	90	1.985
	CL-20-CCNN FF	9.673	12.374	12.443	90.5	91.52	89.25	1.955
	dev %	0.03	4.9	6.8	0.55	1.7	0.8	1.5
γ	Exp^a	13.231	8.17	14.876	90	109.17	90	1.916
	CL-20-CCNN FF	13.409	7.665	15.325	90.62	108.99	89.55	1.955
	dev %	1.34	6.19	3.02	0.69	0.16	0.50	2.0

^{a} experimental values obtained from ref.[S5]

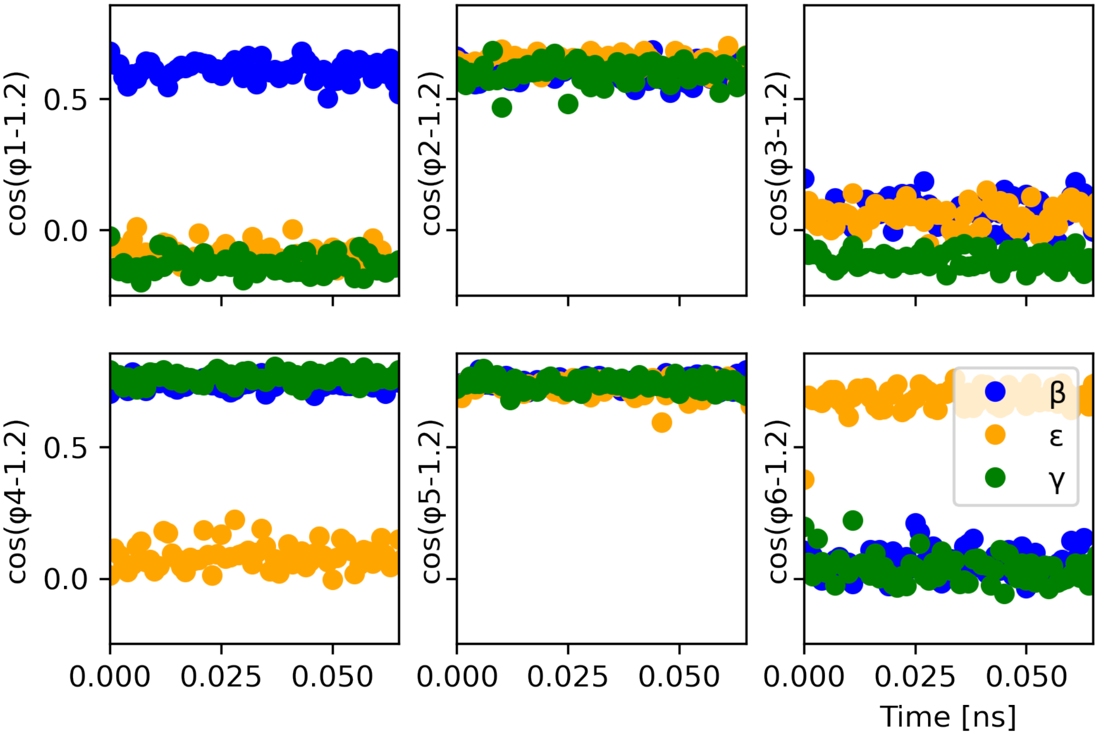

Figure S1: Cosines of the improper angles with an offset phase of 1.2 radians from unbiased 70 ps simulations of isolated CL-20 conformers.

Table S2: Coefficients and weights of each improper angle descriptors ($\cos(\varphi_i - 1.2)$) from MC-HLDA using all six of them.

φ_i	φ_1	φ_2	φ_3	φ_4	φ_5	φ_6
c_i	-0.4712	-0.0417	0.0834	-0.8313	-0.0899	0.2646
$w_i(\%)$	22.21	0.17	0.7	69.11	0.81	7.0

Table S3: Coefficients and weights (w_i) for improper angle descriptors ($\cos(\varphi_i - 1.2)$) from MC-HLDA using only φ_1, φ_4 and φ_6.

CV_i	c_1	c_4	c_6	$w_1[\%]$	$w_4[\%]$	$w_6[\%]$
CV_1	-0.4342	-0.8641	0.2546	18.8	74.7	6.5
CV_2	-0.822	0.565	-0.074	67.6	31.9	0.5

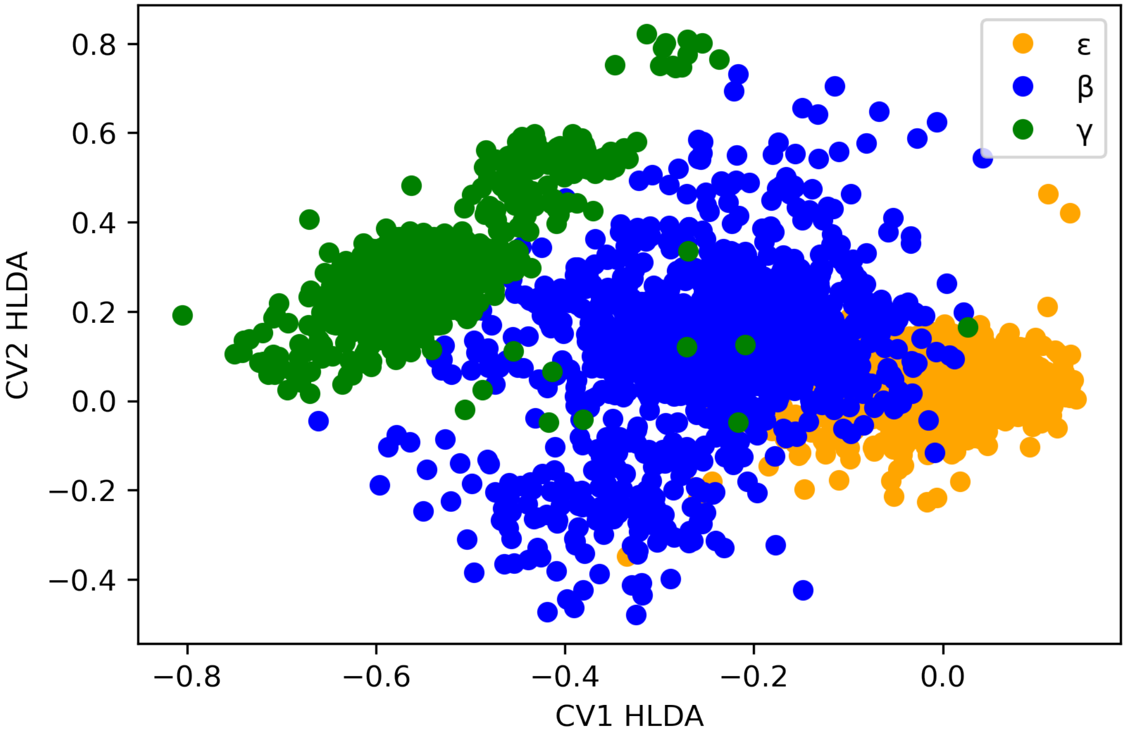

Figure S2: Scatter plot of CL-20 polymorphs in the space of the two MC-HLDA CVs, data obtained from ~ 70 ps unbiased unit-cell simulations.

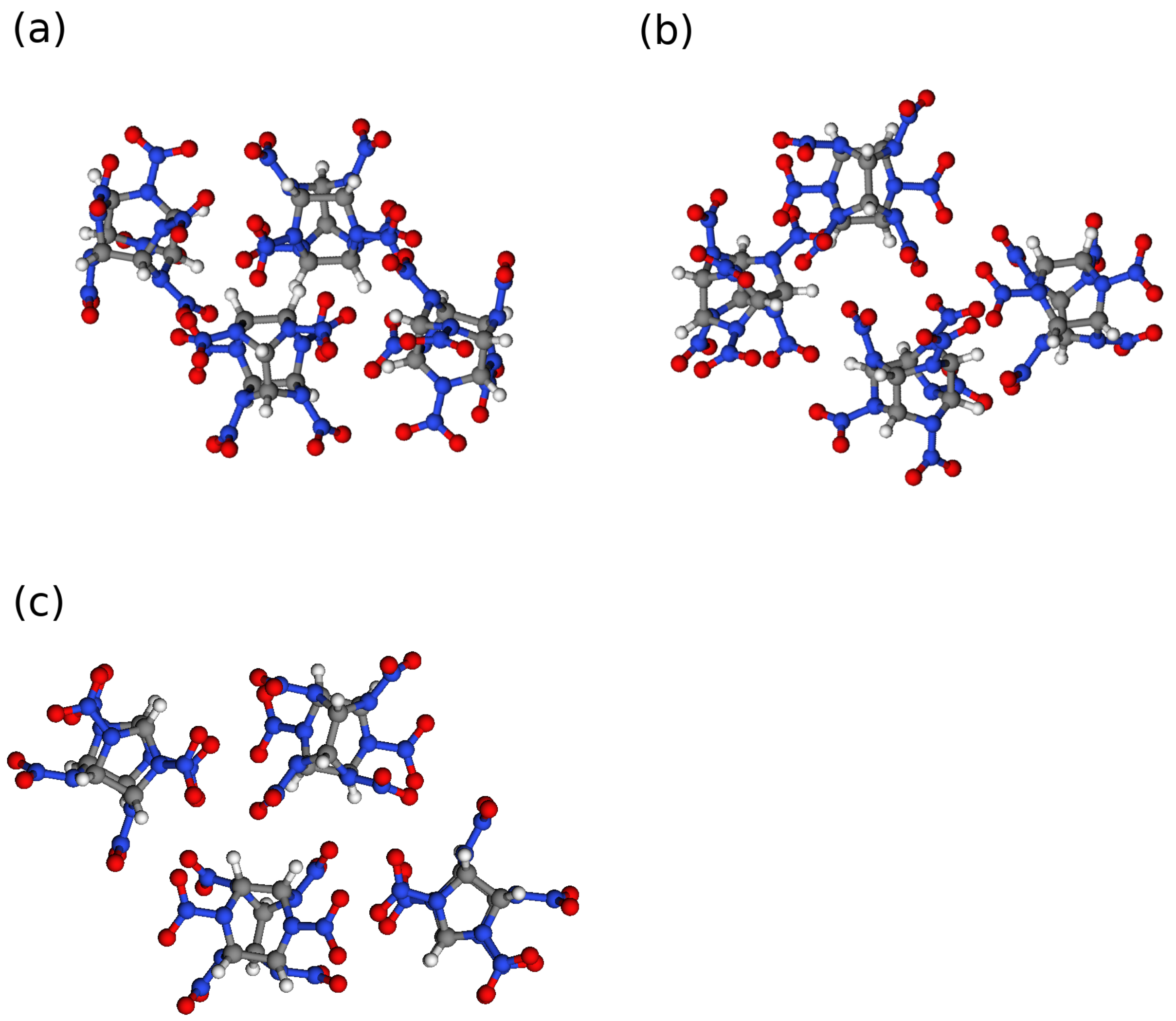

Figure S3: Snapshots of molecular orientation of CL-20 polymorphs during biased simulation of the solid, (a) ϵ-CL-20, (b)γ-CL-20, and (c) β-CL-20.

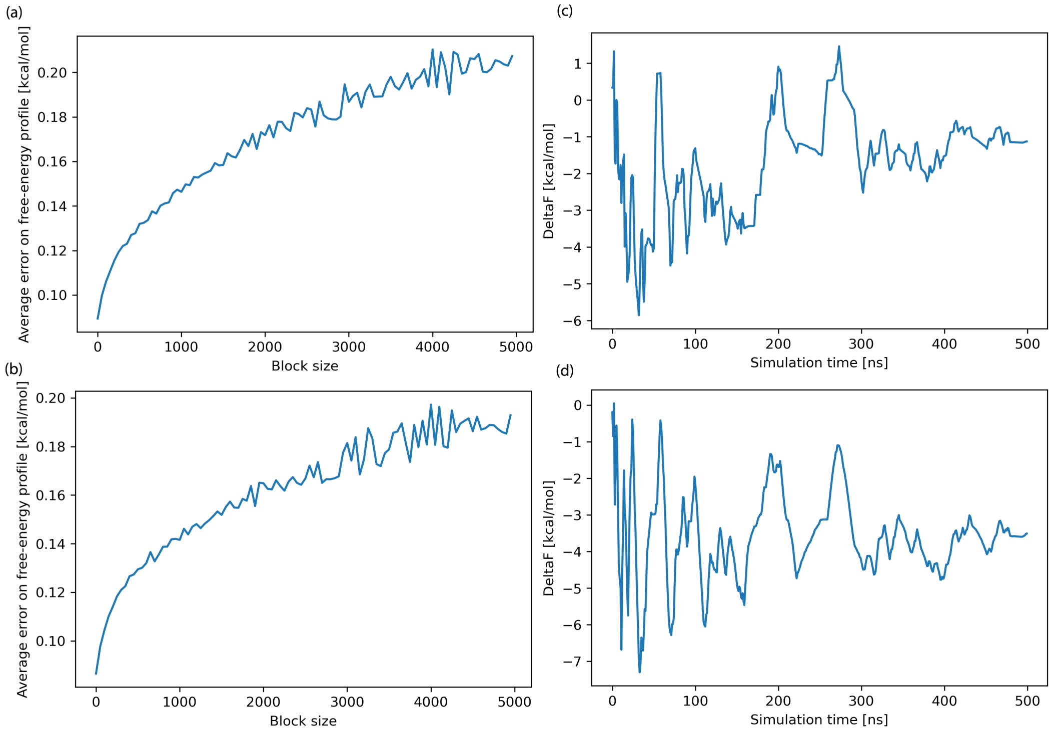

Figure S4: (a) Average error in FES as function of block size using CV1, (b)Average error in FES as function of block size using CV2, (c) energy difference between ϵ- and γ-CL-20 and, (d) energy difference between ϵ- and β-CL-20.

Table S4: Lattice parameters of the CL-20 defect forms

Form	$a[\AA]$	$b[\AA]$	$c[\AA]$	$a[^\circ]$	$b[^\circ]$	$c[^\circ]$
II,IV	8.909	12.2302	13.438	80.421	117.323	94.53
III	7.997	14.304	16.034	120.444	111.43	86.764
γ_{defect}	10.234	13.084	11.356	88	98	89

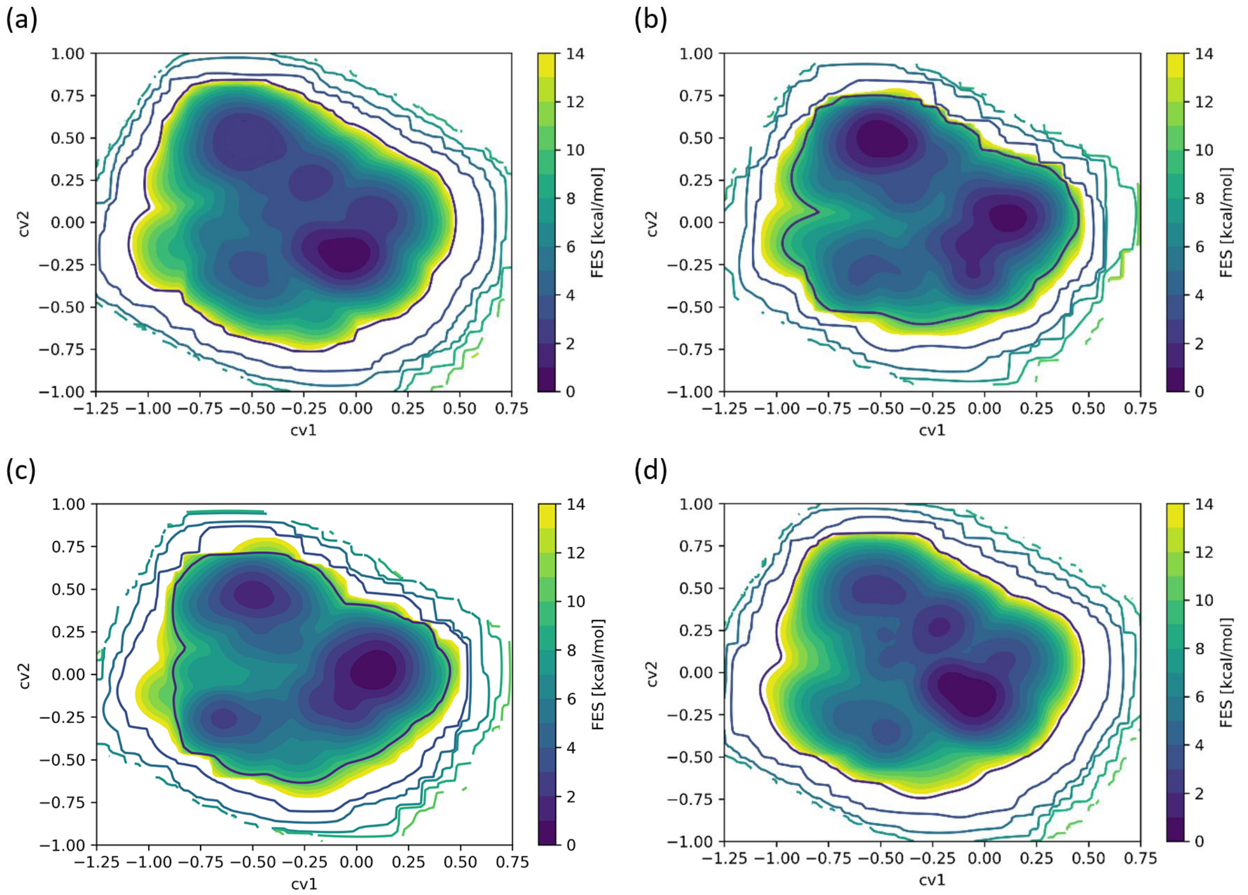

Figure S5: FES of independent simulations with different seeds. Each seed was randomly chosen. The simulations were run for $150 - 500\,ns$.

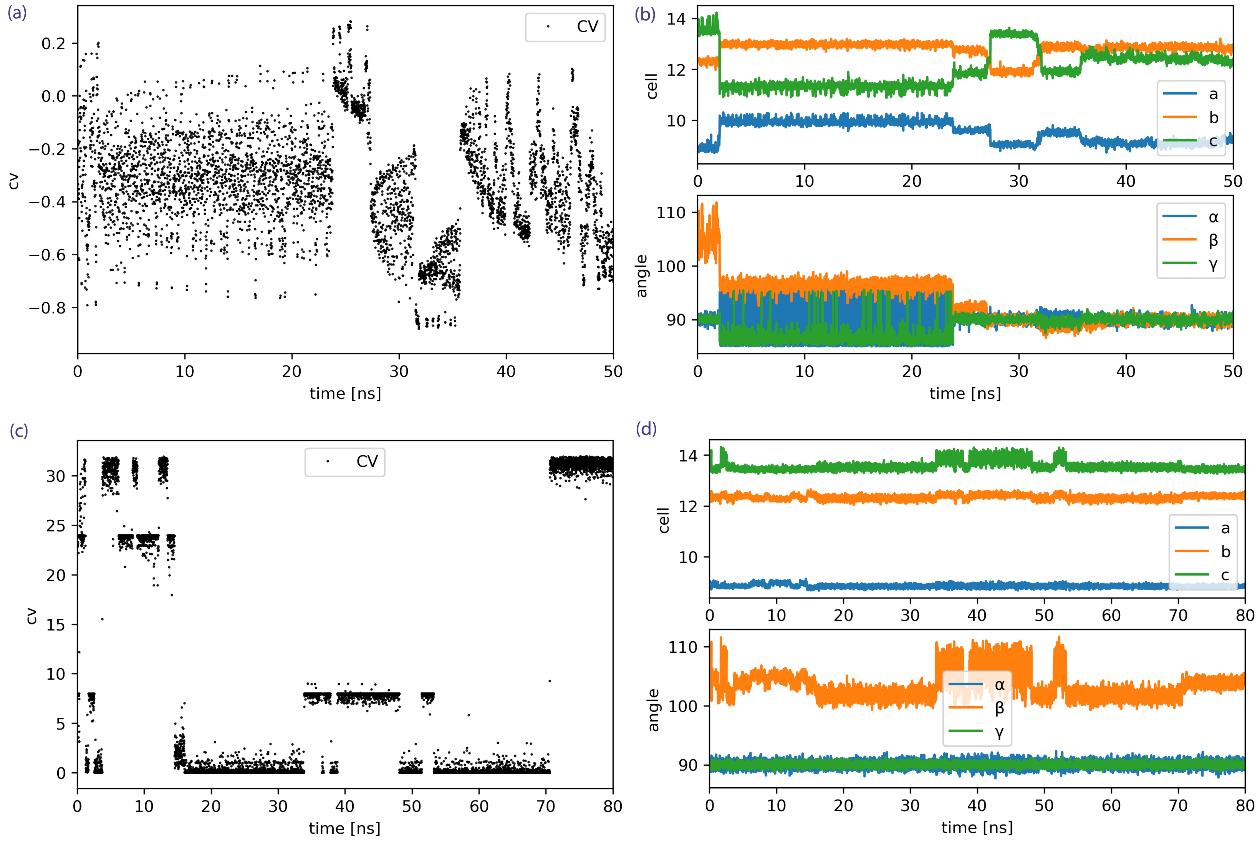

Figure S6: Preliminary supercell 2x2x2 WT-MetaD simulations: (a) CV obtained as an average over the corresponding values of all molecules in the supercell, as in the main text, and (b) cell parameters of the supercell using the average CV. Transitions from epsilon to a defect, gamma, and then beta phases are observed. (c) CV based on a switching function counting the number of epsilon molecules in the supercell, and (d) cell parameters of the supercell using the switching function CV. A transition from the epsilon to the gamma phase is observed.

Table S5: Lattice parameters and densities of CL-20 polymorphs from DFT calculations

$Polymorphs$	$a[\AA]$	$b[\AA]$	$c[\AA]$	$a[^\circ]$	$b[^\circ]$	$c[^\circ]$	$\rho[gr\,cm^{-3}]$
$\beta_{experiment}$	9.676	13.006	11.649	90	90	90	1.985
β_{DFT}	9.515	13.063	11.419	90	90	90	2.048
$\beta_{error}[\%]$	1.66	0.44	1.97	0	0	0	3.2
$\gamma_{experiment}$	13.231	8.170	14.876	90	109.15	90	1.916
γ_{DFT}	13.085	8.170	14.675	90	109.012	90	1.96
$\gamma_{error}[\%]$	1.1	0	1.35	0	0.13	0	2.3
$\epsilon_{experiment}$	8.852	12.556	13.386	90	106.82	90	2.044
ϵ_{DFT}	8.805	12.474	13.259	90	106.34	90	2.08
$\epsilon_{error}[\%]$	0.53	0.65	0.95	0	0.45	0	1.8

We applied DFT to investigate the stability of all observed intermediate and defect forms. The γ-form with slightly different lattice parameters observed in our MetaD simulations did not converge during cell relaxation. Thus, it is possible this form is an artifact of the applied force field.

Table S6: Lattice parameters and densities of the stable CL-20 intermediate form (I) from DFT calculations

$Form$	$a[\mathring{A}]$	$b[\mathring{A}]$	$c[\mathring{A}]$	$a[°]$	$b[°]$	$c[°]$	$\rho[gr\,cm^{-3}]$
$I_{MD-initial}$	9.128	12.186	14.578	80.421	117.323	94.53	2.015
I_{DFT}	8.851	12.378	14.269	81.336	114.818	94.323	2.0728
$I_{error}[\%]$	3.04	1.57	2.12	1.14	2.14	0.22	2.83

References

(S1) Shinoda, W.; Shiga, M.; Mikami, M. Rapid Estimation of Elastic Constants by Molecular Dynamics Simulation under Constant Stress. *Physical Review B* **2004**, *69*, 134103.

(S2) Tuckerman, M. E.; Alejandre, J.; López-Rendón, R.; Jochim, A. L.; Martyna, G. J. A Liouville-operator Derived Measure-preserving Integrator for Molecular Dynamics Simulations in the Isothermal–isobaric Ensemble. *Journal of Physics A: Mathematical and General* **2006**, *39*, 5629.

(S3) Parrinello, M.; Rahman, A. Polymorphic Transitions in Single Crystals: A New Molecular Dynamics Method. *Journal of Applied Physics* **1998**, *52*, 7182.

(S4) Martyna, G. J.; Tobias, D. J.; Klein, M. L. Constant Pressure Molecular Dynamics Algorithms. *The Journal of Chemical Physics* **1998**, *101*, 4177.

(S5) Nielsen, A. T.; Chafin, A. P.; Christian, S. L.; Moore, D. W.; Nadler, M. P.; Nissan, R. A.; Vanderah, D. J.; Gilardi, R. D.; George, C. F.; Flippen-Anderson, J. L. Synthesis of Polyazapolycyclic Caged Polynitramines. *Tetrahedron* **1998**, *54*, 11793–11812.
